# Real-time observation of domain fluctuations in a two-dimensional magnetic model system

**DOI:** 10.1038/ncomms7832

**Published:** 2015-04-22

**Authors:** M. Kronseder, T. N. G. Meier, M. Zimmermann, M. Buchner, M. Vogel, C. H. Back

**Affiliations:** 1 Physics Department, Universität Regensburg, Universitätsstr. 31, 93040 Regensburg, Germany

## Abstract

Domain patterns of perpendicularly magnetized ultra-thin ferromagnetic films are often determined by the competition of the short range but strong exchange interaction favouring ferromagnetic alignment of magnetic moments and the long range but weak antiferromagnetic dipolar interaction. Detailed phase diagrams of the resulting stripe domain patterns have been evaluated in recent years; however, the domain fluctuations in these pattern forming systems have not been studied in great detail so far. Here we show that domain fluctuations can be observed in ultra-thin two-dimensional ferromagnetic Fe/Ni/Cu(001) films with perpendicular magnetization in the stripe domain phase. Non-stroboscopic time-resolved threshold photoemission electron microscopy with high temporal resolution allows analysing the dynamic fingerprint of the topological excitations in the nematic domain phase. Furthermore, proliferation of domain ending defects in the vicinity of the spin reorientation transition is witnessed.

## Abstract (web summary)

The walls between domains in magnetic thin films close to a spin reorientation transition fluctuate owing to thermal excitation. Here Kronseder *et al*. image these fluctuations in iron/nickel/copper films using time-resolved threshold photoemission electron microscopy.

## Graphical abstract

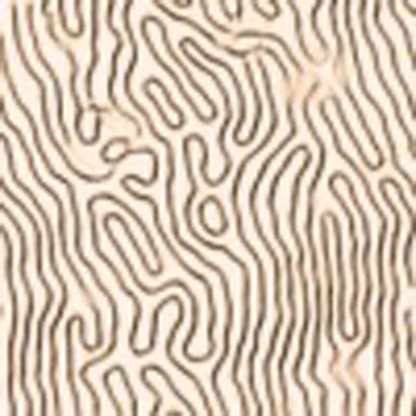

Competition between attractive short range and repulsive long range interactions may lead to intricate pattern formation that can be observed in diverse complex systems^1^,^2^,^3^,^4^. In such systems, a strong but short range interaction favours a uniform phase, but is in competition with a much weaker but long range interaction that favours mixing of phases. This competition leads to energetic frustration and the evolution of complex domain patterns demonstrated by phase separation on mesoscopic or even macroscopic length scales. Patterns transform as a function of external parameters such as temperature; however, astonishingly little is known about the mechanisms of pattern transformation and the role of fluctuations even in rather simple model systems. In ferromagnetic systems, two large material classes displaying stripe and bubble type patterns have been intensively investigated. Magnetic garnets are ferrimagnetic systems that have been model systems for complex pattern evolution in three dimensions^2^. Even more interesting from a theoretical point are ultra-thin ferromagnetic films since here the influence of fluctuations is strongly enhanced^5^,^6^,^7^,^8^,^9^,^10^,^11^,^12^,^13^.

In the prototypical systems Fe/Cu(001) and Fe/Ni/Cu(001), many modulated phases such as stripe phases, labyrinthian phases and bubble phases have been discovered and have been classified in appropriate phase diagrams^3^,^14^,^15^,^16^. The universality of the pattern formation process allows mapping these phase diagrams to those of other quasi two-dimensional (2D) systems with competing interactions such as nematic and smectic phases in liquid crystal systems^17^,^18^,^19^,^20^,^21^,^22^,^23^. Despite the vast experimental and theoretical investigations, some important points in the phase diagram of these systems have remained elusive and cannot be addressed using static imaging alone. Attempts have been made to link global temperature-dependent thermodynamic properties such as the magnetic susceptibility to different domain phases^24^,^25^, while other studies have addressed domain wall mobility as a function of temperature^26^,^27^. None of the studies performed so far has been able to combine high temporal and spatial resolution to reveal fluctuations of the order parameter on short time scales.

In the following, we study perpendicular domain phases of the 2D ferromagnetic system Fe/Ni/Cu(001) with high real-time temporal (<1 ms) and spatial resolution (<100 nm)^28^ and focus on the dynamic aspects in the observed domain phases. The transition between individual stripe phases is driven by nucleation and proliferation of topological defects^8^. It has been shown, for example, that the smectic phase supports four types of topological excitations appearing in bound pairs^8^. With rising temperature bound dislocation pairs are predicted to become more abundant and finally melting of the smectic crystal is caused by unbound, proliferating dislocations^8^. In our experiments, we identify the relevant topological defects and reveal their dynamical fingerprints. In particular, we address fluctuations of topological excitations near the transition from an ordered stripe phase to a phase with reduced order. In general, the transformation of phases is often driven by rare events, difficult to capture using slow imaging techniques. By analysing fluctuations of the different topological defects, we reveal their relation to the occurrence of rare events. In fact, we are able to image rare events emanating from topological defects directly.

## Results

### Domain patterns near the spin reorientation transition

**Figure 1:**
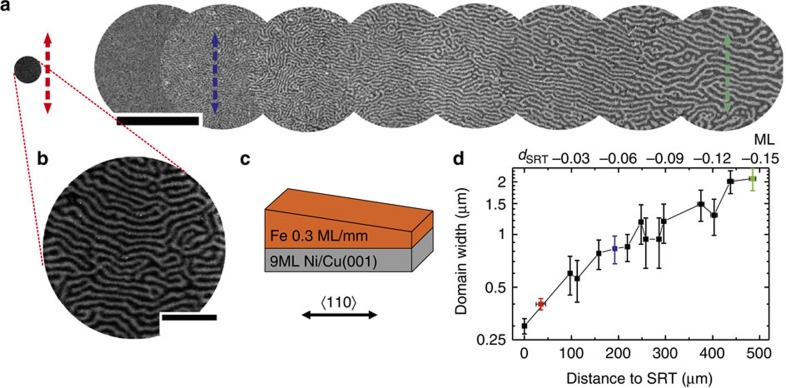
Overview of the domain patterns. (**a**) Magnetic images of the domain pattern evolution. The red, blue and green arrows in **a** mark the positions for the fluctuation analysis. (**b**) The stripe domain pattern maintains its orientational order while approaching the SRT. (**c**) Sketch of a typical wedge-type sample (Fe-wedge on 9 ML Ni/Cu(001), slope of Fe-wedge 0.3 ML mm^−1^). (**d**) Evolution of the domain width as a function of the Fe film thickness in the vicinity of the spin reorientation transition. The error bars arise from the statistical analysis of the domain wall width within each individual PEEM image. Note that the images may have different fields of view. Scale bar, 50 μm (**a**); 5 μm (**b**).

An overview of the observed domain pattern in the vicinity of the spin reorientation transition (SRT) in Fe/Ni/Cu(001) for a typical wedge-type thin-film sample (for sketch see Fig. 1c) is shown in Fig. 1a. The red, blue and green arrows in a mark the positions for the fluctuation analysis shown in Fig. 3. Note that the images are recorded at room temperature and in the as-grown state. The crystallographic axis is indicated in Fig. 1c. Different wedges with the same composition and same or different slopes (see Supplementary Note 1 and Supplementary Fig. 1) have been grown along different crystallographic orientations to confirm that the stripe orientation—if present—solely depends on the crystallographic directions as predicted in refs 29, 30.

In this system, a well-studied SRT occurs at a thickness *d*_SRT_ indicated in Fig. 1d and in close distance to the image shown in Fig. 1b. It is known that the temperature at which the SRT occurs decreases with increasing Fe thickness^14^. For the thickness range studied here, the SRT occurs within the Fe ferromagnetic phase^14^ when measured at room temperature and for the wedge shown in Fig. 1a, *d*_SRT_=*d*_Fe_=1.8±0.3 ML (monolayers). The minimum domain width at the SRT is around 300 nm. Note that we do not focus on the details of the SRT that may be spiral-like^31^, but that we focus on the dynamics of the domain pattern evolution in the perpendicular stripe phase. We would like to point out that an in-plane magnetic contrast can be observed beyond the SRT (shown in Supplementary Fig. 1), which can be distinguished from the perpendicular phase by the formation of larger irregular domains. The domain width shown in Fig. 1a decreases exponentially as a function of distance towards the SRT^14^, and thus with increasing film thickness, see Fig. 1d and Supplementary Fig. 1(c). A similar dependence on sample temperature is observed as well^14^,^28^.

From these types of experimental data, one can in principle determine the predicted domain phases in this system. Similar to the seminal experiments by Vaterlaus *et al*.^32^ and Portmann *et al*.^3^ on the system Fe/Cu(001) and experiments by the Qiu group^14^ performed on Fe/Ni/Cu(001) films and in line with the theoretical predictions by Abanov *et al*.^8^, we observe large domains at lowest thickness (lowest temperature) representing a monodomain phase that is altered to a random domain phase with large disordered domains due to pinning centres (not shown). At higher thickness (higher temperature), this phase is transformed into a stripe domain phase close to the SRT. Note that a smectic phase is characterized by long range directional and positional order, which decays algebraically while in a nematic phase the positional order decays exponentially. Due to the rather small field of view of our experiment, we cannot distinguish these phases unambiguously, whereas the orientational order can be confirmed. Note that according to refs 33, 34, positional order is strongly suppressed in systems with isotropic interactions in 2D, whereas ‘orientational order is more robust'. This may approximately apply to the magnetic system realized in our experiments since the in-plane fourfold anisotropy is rather weak.

**Figure 2:**
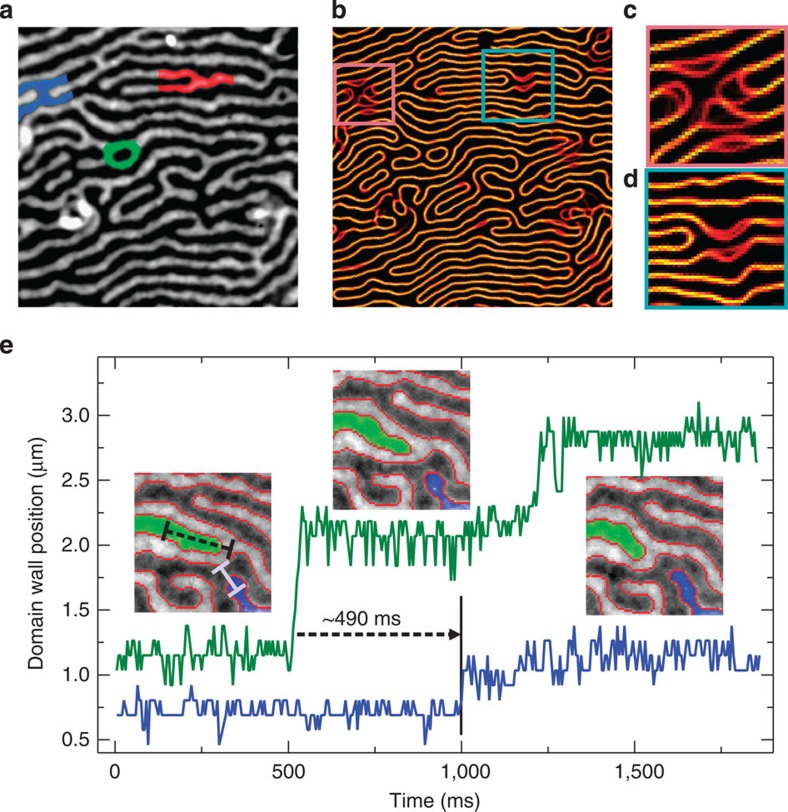
Non-stroboscopic time-resolved imaging. (**a**) Domain pattern close to the SRT with an equilibrium domain width of *w*_D_≈800 nm. An A-type defect is highlighted in red, a C-type defect in blue and a D-type defect is coloured in green. (**b**) The sum of 2,738 individual domain wall images reveals areas with fluctuating domain walls. (**c**) A region with fluctuating domain walls in the vicinity of the C-type defect highlighted in **b**, for the A-type defect this is shown in **d**. Image sizes: (**a**,**b**): 25.8 × 25.8 μm, (**c**): 4.7 × 4.7 μm, (**d**): 5.9 × 5.9 μm; frame rate 200 fps, *T*=295 K. (**e**) An image sequence of the propagation (coloured in blue) and retraction (coloured in green) of two A-type defects. The lines in the first inset represent the linescan regions (black dashed line for the green curve and white line for the blue curve). Image size of the insets 7.9 × 7.9 μm.

According to theory, on raising the thickness (temperature), the smectic phase eventually transforms to the nematic phase mediated by the two types of fluctuations. When the stripe bending energy is smaller than the stripe orientation energy, meandering of the domain walls can be observed that decreases positional order and causes the smectic crystal to lose its orientational rigidity^8^, sometimes called transverse instability (the elastic energy of the stripe domains is illustrated in Supplementary Fig. 2 and discussed in Supplementary Note 2). Furthermore, the smectic phase supports four types of topological defects^8^,^27^,^32^, see Fig. 2a. Recently, these excitations have been observed in the Pt/Co/Pt system and have been imaged with a temporal resolution of 0.2 frames per second^27^. In the smectic phase, these dislocations are predicted to be bound^8^. As thickness (temperature) is increased, their number increases and algebraic positional order is finally destroyed by proliferation of unbound dislocations.

In most of our experiments, an orientationally ordered phase (in the following called nematic) is followed by a completely disordered phase before the SRT occur, as shown for instance in Supplementary Fig. 1. Alternatively, a re-entrant transition into an ordered stripe phase has been observed^3^, which we can confirm in some cases. At this point, we emphasize that while the different phases are characterized by orientational and positional order independent of the width of the domains, the domain width itself continuously decreases with rising thickness (temperature) following an unambiguous dependence^14^, see Fig. 1d, Supplementary Note 1 and Supplementary Fig. 1.

### Thermal fluctuations of domain walls

In the following, we will demonstrate that a non-stroboscopic imaging technique with high temporal resolution can be used efficiently to search for the dynamical fingerprint of the stripe domain phase of this 2D magnetic model system. This has previously only been possible by monitoring average quantities such as the ac-susceptibility^24^,^25^. In microscopic imaging techniques, fluctuations have appeared as blurry regions or have been evidenced by changes in the stripe position or density between individual image line scans^3^,^14^,^27^.

The concept of the approach is straight forward. In a perfect smectic stripe phase, for example, it is expected that fluctuations are suppressed due to geometric constraints. In a fully disordered phase or a smectic/nematic phase with topological defects, on the other hand, the domain patterns are less constrained and one might expect fluctuations to be more pronounced. Our unique experimental method allows us to record images with exposure times as short as 450 μs enabling the analysis of non-stroboscopic real-time movies of the fluctuating domains as a function of effective temperature.

Figure 2a shows an overview image (image size 25.8 × 25.8 μm^2^, images recorded at 200 frames per second) in the nematic stripe domain phase with pronounced defects marked in colour. The Fe-wedge/(9 ML)Ni/Cu(001) sample is the same as shown in Fig. 1. We can clearly identify all the four types of defects (A/B-type: dislocations inserted into black/white domains (red colour), C-type: bridge defect (blue colour) and D-type: island (green colour)) as classified in ref. 8. The image shown here corresponds to the average of an image stack containing 2,738 images, which corresponds to a domain image recorded with ∼14 s exposure time. To visualize the characteristic fluctuations, we obey the following protocol. First an image sequence using a certain frame rate of up to 2,200 fps (corresponding to a single image exposure time of 450 μs) is recorded (200 fps in the case of Fig. 2a). Subsequently, the single images are transformed into line images allowing identification of the domain boundaries, see Supplementary Fig. 3 and Supplementary Note 3. In Fig. 2b, we overlay the obtained images again. The colour coding refers to the probability for the domain walls occurring in certain positions. In Fig. 2c,d, fluctuations are visualized for the C-type and the A-type defect highlighted in Fig. 2a.

### Domain-width-dependent strength of thermal fluctuations

**Figure 3:**
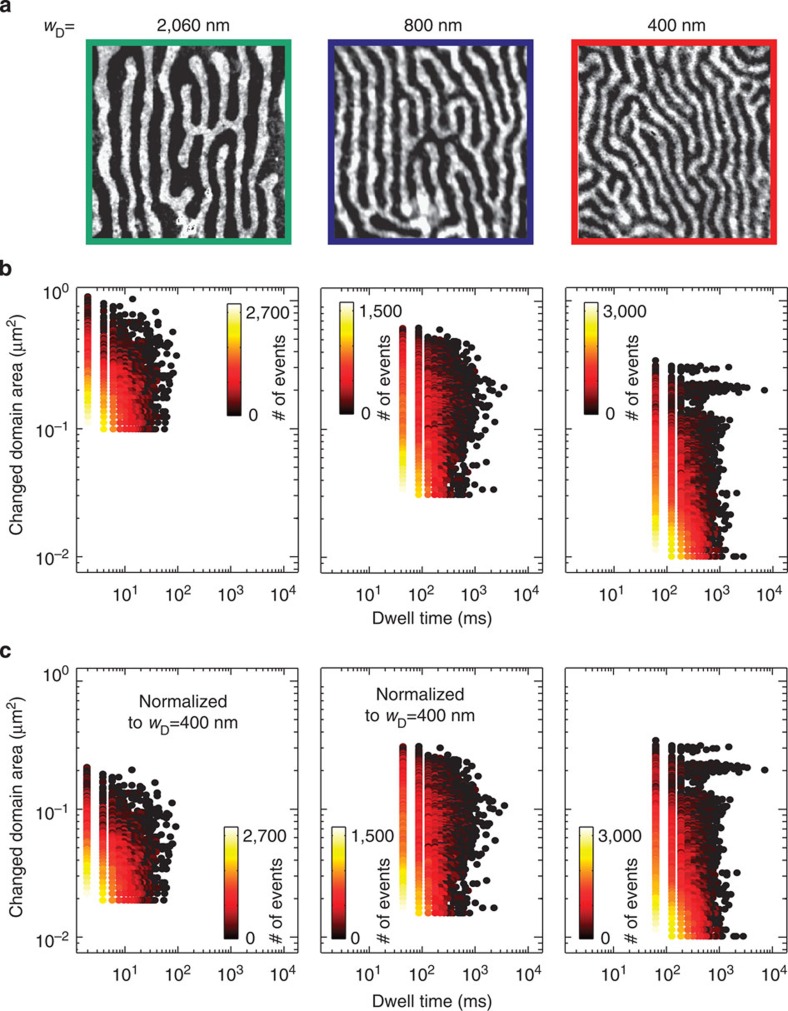
Influence of thermal energy on the stripe domain pattern with different width. (**a**) Nematic domain patterns with domain widths of *w*_D_≈2,060, 800 and 400 nm recorded at room temperature. (**b**) Evaluation of the domain fluctuations with respect to the dynamic change of the domain area as well as dwell time. The corresponding frame rates are 500, 24 and 16.4 fps and the total recording times for the data sets are 5.48, 114 and 119 s, respectively. In **c**, the changed domain size is normalized to the smallest domain width, that is *w*_D_≈400 nm. The colour code indicated in **b** shows the total number of recorded events.

To evaluate the observed fluctuations, we calculate the local change of the domain area and register the time the fluctuating area remains in the new state (dwell time) before it jumps back into its original configuration or into a new state defined by an area change of at least 5–10 pixels depending on the field of view and corresponding resolution. We first apply this evaluation method to the nematic domain phase with varying domain width. Typical domain images with decreasing domain width are shown in Fig. 3a. The colours of the boxes in Fig. 3a are consistent with the coloured arrows in Fig. 1a, indicating the position on the wedge. According to Fig. 1a,d, the decreasing domain width in the series evaluated here is due to the increasing Fe film thickness (corresponding to increasing temperature). Thus, in this series, the SRT is approached and the nematic phase has to transform into a less ordered phase. Consequently, this phase is more and more destabilized by the appearance and proliferation of topological defects. Note that orientational order decreases with decreasing domain width, see Supplementary Fig. 4 and Supplementary Note 4. As can be seen by the total number of events (see colour coding indicated in Fig. 3b), an enormous amount of fluctuations can be observed. This is a surprising and important finding since it may explain why the determination of the domain wall width has been impossible in this and similar systems when using slow static imaging techniques. In addition, the continuous spectrum of the fluctuations, in particular, with respect to the change of the domain area, indicates a high density of different available domain states. We observe that the maximum value of the fluctuating domain area decreases with decreasing domain width, see Fig. 3b. This signifies that the effect of thermal energy, which is the same for all the three cases since all movies are recorded at ambient temperature, is different and depends primarily on the domain width. To explain this behaviour, we consider the following. One can assume that the density of external domain wall pinning sites such as structural imperfections is similar for the domain configurations shown in Fig. 3a, since the measurements are conducted on the same sample. However, both the dependence of the local energy landscape on the domain pattern itself as well as the glassiness due to the uniform frustration^35^ arising from the competition between long range dipolar and short range exchange interaction lead to the emergence of metastable states separated by energy barriers, which depend on the details of the domain patterns as well as on their most dominant parameter, the domain width. Consequently, the fingerprint of the fluctuations becomes domain width dependent. To evaluate the relative strength of thermal fluctuations for the different domain patterns with respect to the domain width the data is rescaled. Figure 3c shows the same data as Fig. 3b, except that the changed domain areas for the domain widths *w*_D_≈2,060 nm and *w*_D_≈800 nm are rescaled to the case of *w*_D_≈400 nm. This figure reveals that the relative maximum of the fluctuating domain area in fact increases slightly with decreasing domain width, whereas the absolute maximum fluctuating domain area decreases as shown in Fig. 3b. Thus, on average, thermal energy induces larger domain wall jumps in a domain pattern with wide domains than in a narrow domain pattern. However, in relative terms, the impact of thermal energy is larger for narrow domains, which means that the strength of thermal fluctuations increases with decreasing domain width. This behaviour can be related to the different rigidities of the domain patterns.

### Fluctuation properties of individual topological defects

**Figure 4:**
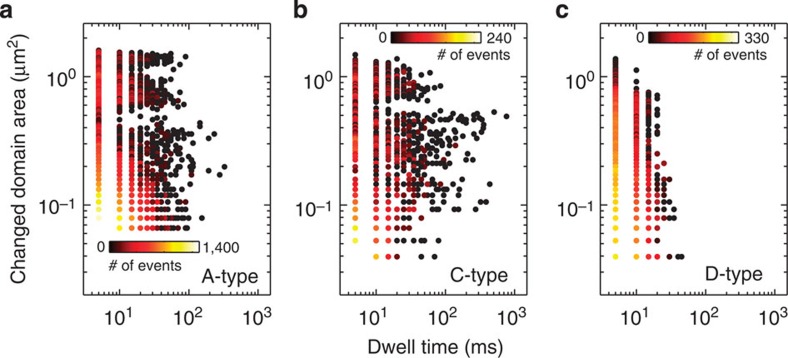
Fluctuations of the topological defects. The fluctuation properties with respect to changed domain area as well as dwell time are shown for A-type defects in **a**, C-type defects in **b** and D-type defects in **c**. The average domain width is 800 nm. The evalution of the changed domain area versus dwell time was performed within an area of 60 μm in diameter on the sample, where the exposure time for a single image was 5 ms (200 fps) and the investigated time span was 13.98s in total.

Next, we take a closer look at the dynamical fingerprint of topological defects such as the ones marked in Fig. 2a, since it is the increase of their number that ultimately drives the transition into a more disordered state. Figure 2a represents a typical region under investigation, however, the data summarized in Fig. 4 contains more defects than shown in the detail image in Fig. 2a. Cumulated images (frame rate 200 fps, 2,738 images) are shown for a particular C-type defect as well as for an A-type defect in Fig. 2c,d. Significant differences in the fluctuations can be observed at first glance; however, only the statistics collected in the experiments reveals the dynamical fingerprint. The results are summarized in Fig. 4. Clearly, the isolated D-type defects show reduced fluctuations when compared with the A-type and C-type defects. In particular, fluctuations able to drive large changes in the domain pattern, for example, large fluctuating areas and long dwell times are suppressed. This observation is easy to interpret since the enclosing domains lead to both, isolation of the D-type defects as well as to strong geometrical constraints suppressing fluctuations. In contrast, both A-type and C-type defects display large area fluctuations as well as long dwell times. A significant contribution at largest area changes is observed for A-type defects and we postulate that these defects ultimately drive the transition. This can be understood by considering that typically A-type defects are not observed in weakly bound pairs in our experiments, since the data is taken reasonably close to the transition. On the other hand, C-type defects do display large area changes as well, but the fluctuating motion of the domains is highly correlated, related to the fact that C-type defects represent two strongly bound A-type defects. Thus, typically these defects react to each other in the sense that their motion is dependent on each other and can be even synchronous, in both cases inhibiting the propagation of a defect, see Supplementary Fig. 5, Supplementary Fig. 6 and Supplementary Note 5. In stark contrast, unbound A-type defects eventually propagate leading to domain pattern transformation. Note that A-type defects may emerge either from unbinding of a C-type defect or from transverse instabilities, in the form of rare events. This is exactly what has been proposed by Abanov *et al*.^8^: the long range positional order in the smectic phase decays algebraically due to long-wavelength excitations as well as pairs of dislocations, which become unbound and proliferate at increased temperatures. Hence, the conclusion can be drawn that mostly A-type defects are responsible for the transformation of patterns. We have in fact captured such a rare event within the same image area, displayed in detail in Fig. 2e. The images show the growth of the A-type defect coloured in blue that is linked to the reduction of the A-type defect coloured in green. In fact, the reduction of the green domain triggers the propagation of the blue domain. The response time, that is, the time delay for the response of the blue domain to the retraction of the green domain is about 490 ms. Within this time period, the black and white domains in the immediate vicinity of the blue and green coloured domains remain stationary with an increased domain width. This unfavourable larger domain width acts as the driving force for the propagation of the blue domain transforming the pattern to a denser nematic phase (for the movie see Supplementary Movie 1).

In conclusion, we have imaged fluctuations of the domain patterns in the vicinity of a spin reorientation transition for the first time with high temporal and spatial resolution. We identify the relevant topological defects and evaluate their dynamical signatures. We conclude that in the particular striped domain phase investigated here, A-type defects drive the phase transition into a less ordered phase. The presented data may prove to be of importance for the analysis of phase transitions in 2D systems. Furthermore, the recorded movies can be viewed as experimental realizations of extremely large scale Monte Carlo simulations of a truly 2D system and may allow to shed light on finite size effect problems often encountered in these simulations.

## Methods

### Sample preparation

The Fe/Ni/Cu(001) samples are prepared using a well-established protocol. We first prepare the Cu(001) single crystal by cycles of soft sputtering and annealing until sharp reflection high-energy electron diffraction spots are visible. The magnetic films are evaporated at a rate of 0.4 ML min^−1^ in ultra-high vacuum with a base pressure better than 2 × 10^−10^ mbar and a pressure during evaporation <3 × 10^−10^ mbar. During evaporation reflection high-energy electron diffraction oscillations are monitored confirming layer-by-layer growth and serving as an accurate thickness calibration. No contamination of the films is visible in X-ray photoemission spectroscopy.

### Imaging technique

To be able to perform fast non-stroboscopic imaging with large magnetic contrast and high spatial resolution, we revert to the recently discovered contrast mechanism of magnetic circular dichroism in threshold photoemission applied to a photoemission electron microscope. The illumination unit comprises a 405 nm (3.06 eV) laser diode with up to 600 mW, typically set to ∼(100−200) mW, a linear polarizer and a rotatable quarter-wave plate. Since the work function of Fe and Ni is around ∼5 eV, Cs adatoms were deposited before PEEM operation to lower the work function and, hence, to increase the photocurrent.

## Author contributions

C.H.B. and M.K. planned the experiment. M.K. performed the experiments. C.H.B. and M.K. analysed the data. T.N.G.M., M.B., M.V. and M.Z. helped with the data analysis. C.H.B and M.K. wrote the manuscript.

## Additional information

**How to cite this article:** Kronseder, M. *et al*. Real-time observation of domain fluctuations in a two-dimensional magnetic model system. *Nat. Commun*. 6:6832 doi: 10.1038/ncomms7832 (2015).

## Supplementary Material

Supplementary Information Supplementary Figures 1-6, Supplementary Notes 1-5 and Supplementary References

Supplementary Movie 1 The Supplementary Movie 1 shows the fluctuating domains of the domain configuration shown in Fig. 2 of the main text and fluctuations in the vicinity of topological defects. Also shown is the evolution of the A-type defect discussed in the manuscript, and the decay of a C-type defect driven by thermal fluctuations and leading to the proliferation of A-type defects.
