# Supplementary material for: Real-time observation of domain fluctuations in a two-dimensional magnetic model system
Source: Nat Commun. 2015 Apr 22;6:6832. doi: 10.1038/ncomms7832 (PMC4423231; doi:10.1038/ncomms7832)
Supplement: Supplementary Information — Supplementary Figures 1-6, Supplementary Notes 1-5 and Supplementary References [file ncomms7832-s1.pdf]

Supplementary Figure 1

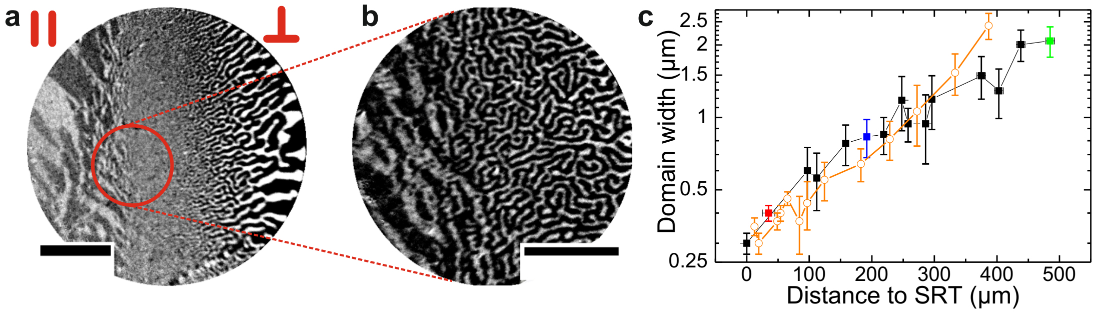

Supplementary Figure 1: **Domain evolution in the vicinity of the spin reorientation transition** (a) Domain evolution for a Fe/Ni/Cu(001) system with a Fe-wedge with slope of 2.1 ML mm^{-1} in the vicinity of the SRT. The irregular domains on the left hand side represent the in-plane phase whereas the right hand side shows the perpendicular phase. The magnified image (b) shows the domain evolution directly at the SRT. The narrowest perpendicular domains have a width of ∼300 nm. Note that the stripe orientation is ∼45° with respect to the wedge orientation, which is along a ⟨110⟩ crystallographic axes. (c) shows the evolution of the domain width in the perpendicular phase as a function of distance from the SRT. The orange open circles correspond to the domain width evolution shown in (a) and (b) scaled to the different slope of the Fe-wedge shown in Fig. 1(a) of the main manuscript (black squares). The error bars are given by the selected field of view and the amount of domains within. Scalebar in (a) 25 µm, (b) 10 µm.

Supplementary Figure 2

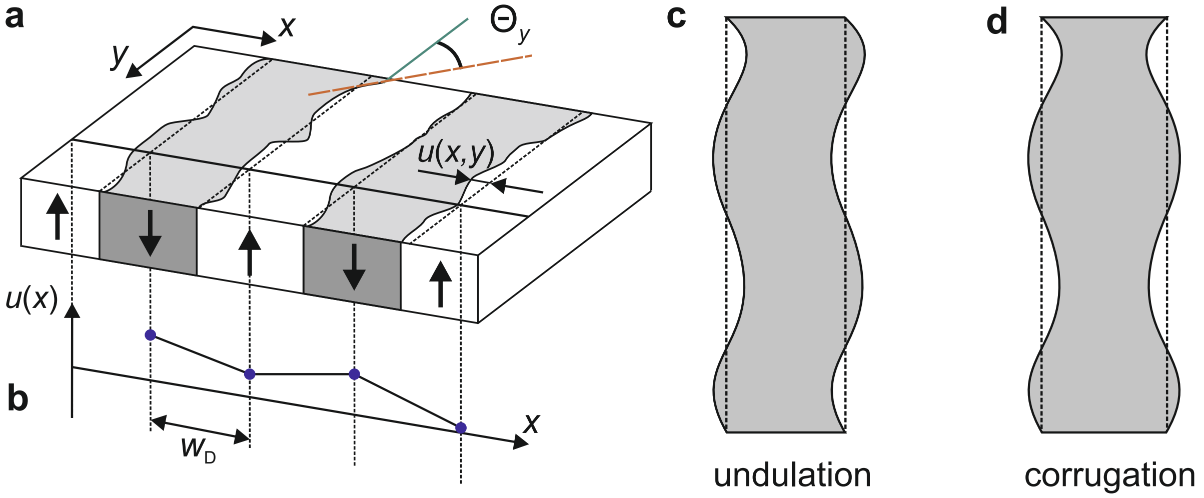

Supplementary Figure 2: **Illustration of thermal excitations of the stripe domain phase** (a) Thermal fluctuations lead to local deviations $u(x, y)$ of the domain walls from the ideal position, i.e. the ground state (dashed line) in the stripe phase [3, 4]. Since the local deviation $u(x)$ is a discrete function, the partial derivative of $u(x, y)$ with respect to x has to be calculated as shown in (b). The two main deviations from the ground stripe state are undulations (c) and corrugations (d).

Supplementary Figure 3

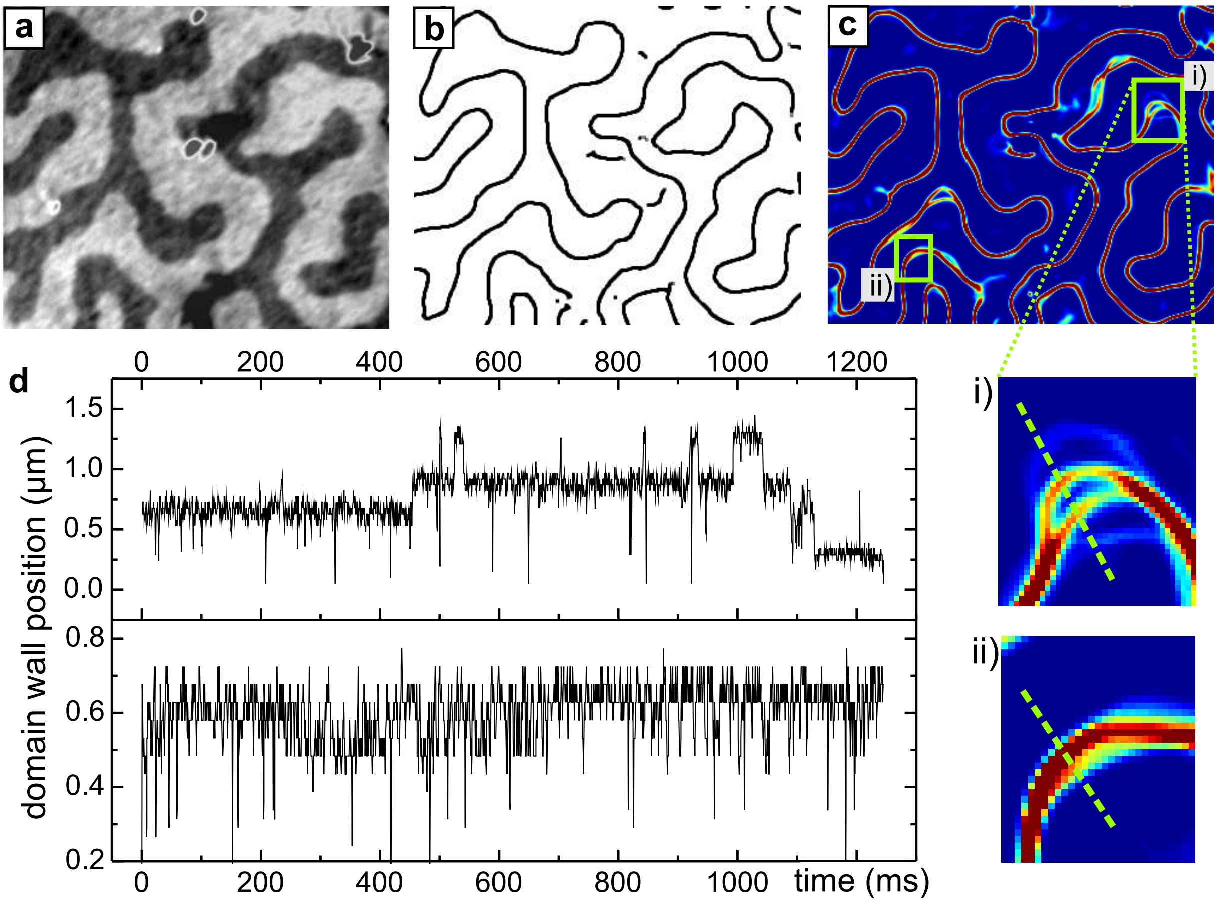

Supplementary Figure 3: **Illustration of the data teatment and analysis** The method to extract the domain wall motion from a sequence of images, such as shown in (a), comprises the realignment of the images as well as the domain wall identification, shown in (b). The summation of all domain wall images (2738) reveals regions in the domain pattern exhibiting large fluctuations, as shown in (c). The time evolution of the individual domain walls can then be extracted by linescans across domain walls for all chronologically ordered images, as shown in (d). In case that the algorithm for the domain wall identification has failed, a spike in the graph appears. (FOV : $15.4 \times 12.8 \mu$m, FR = 2200fps, T = 21°C.)

Supplementary Figure 4

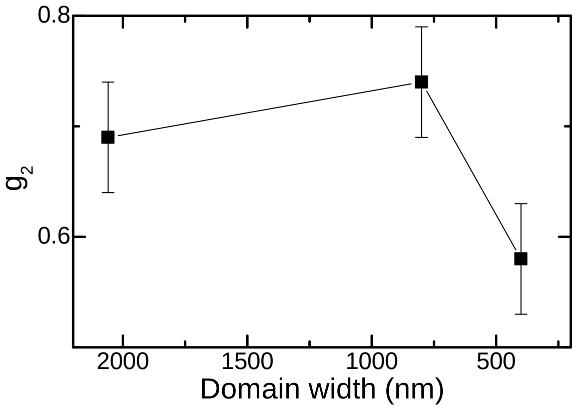

Supplementary Figure 4: **Evaluation of orientational order** The evaluation of the orientational order parameter g_2 is shown for the domain patterns shown in Fig. 3 (a) of the main manuscript.

Supplementary Figure 5

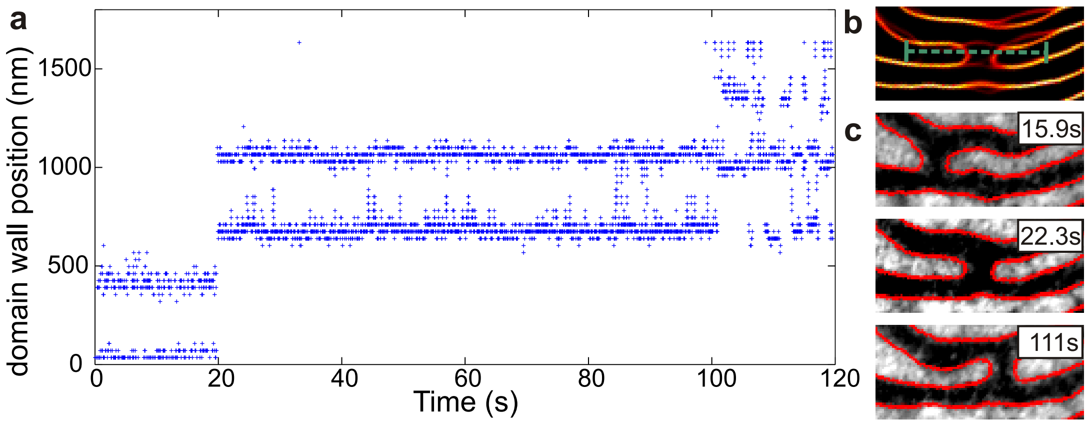

Supplementary Figure 5: **Illustration of the thermal excitation of a C-type defects I** The synchronous, in-phase fluctuation of the two endings of a C-type defect is presented for a nematic domain pattern with an equilibrium domain width of $w_D \approx 400$ nm. (a) shows the result of the temporal evolution of the linescan across both domain endings, as shown by the green dashed line in (b). The domain width of the black domain is kept constant. For times exceeding ~ 100 s the fluctuations are too rapid to be resolved. (b) shows the summarized image of all domain wall images. Snapshots of the individual positions are shown in (c). (FOV : (b)-(c) $3.5 \times 1.6 \mu m$, FR = 16.4fps, T = 21°C)

Supplementary Figure 6

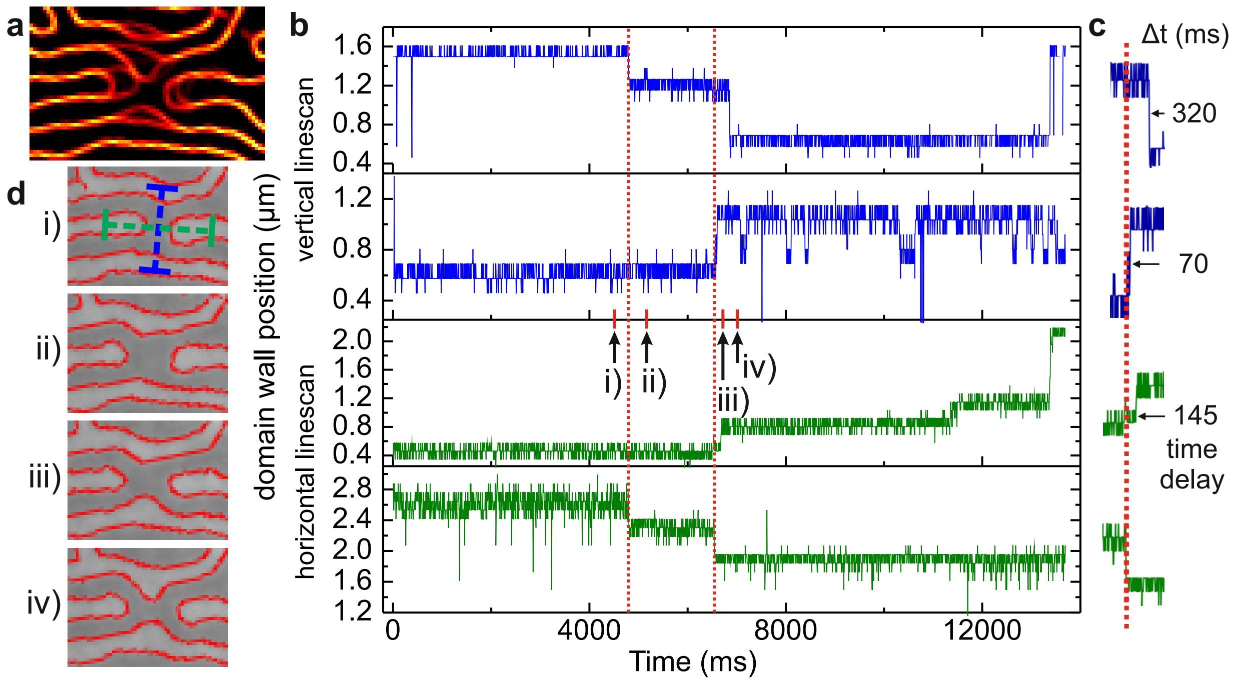

Supplementary Figure 6: **Illustration of the thermal excitation of a C-type defects II** The second possibility for fluctuations of a C-type defect is presented. (a) shows the summarized image. The temporal evolution of the domain walls along the blue, dashed line in (a), is shown in the upper two panels in (b). For the green, dashed line, this is shown in the lower panels, which represent the fluctuations of the domain endings, i.e. the C-type defect. The red dashed lines mark the jumps of the left domain ending. (c) shows the linescan region in the vicinity of the second jump of the left domain ending. Snapshots of the individual stages are presented in (d). (FOV (a) $7.9 \times 5.2\mu$m, (d) $6.7 \times 4.9\mu$m, FR = 200fps, T = 21°C)

Supplementary Note 1
Domain Width Dependence on d, T and K

The basis for pattern formation in two dimensional ferromagnetic systems is the interplay between various magnetic interactions, where the ratio between uniaxial anisotropy λ, dipolar energy Ω and exchange energy Γ, plays the decisive role. Following Kashuba and Pokrovsky [1], for a two-dimensional ferromagnet and a normalized magnetization vector $\mathbf{m}(\mathbf{r}) = \frac{\mathbf{M}}{|\mathbf{M}|}$ at site $\mathbf{r}$ the Hamiltonian reads

$$H = \frac{1}{2}\Gamma(T)\int \mathrm{d}r^2\,[\nabla\cdot\mathbf{m}(\mathbf{r})]^2 - \lambda(T)\int \mathrm{d}r^2\, m_z^2(\mathbf{r}) - \mu_0\int \mathrm{d}r^3\,\mathbf{M}\cdot\mathbf{H}_{ext} + \frac{1}{4\pi}\Omega(T)\iint \mathrm{d}r_1^2\,\mathrm{d}r_2^2 \frac{\mathbf{m}(\mathbf{r}_1)\cdot\mathbf{m}(\mathbf{r}_2) - 3[\hat{\mathbf{r}}_{12}\cdot\mathbf{m}(\mathbf{r}_1)][\hat{\mathbf{r}}_{12}\cdot\mathbf{m}(\mathbf{r}_2)]}{|\mathbf{r}_1-\mathbf{r}_2|^3}\,, \quad (1)$$

where Ω is the strength of the dipole interaction, Γ is the exchange energy, λ is the effective single-ion anisotropy constant, and $\hat{\mathbf{r}}_{12} = \frac{\mathbf{r}_1-\mathbf{r}_2}{|\mathbf{r}_1-\mathbf{r}_2|}$. Ω, Γ and λ are thickness as well as temperature dependent and the domain width w_D in the system with perpendicular magnetization described by Eq. (1) also depends on these parameters. In fact, the dependence of the equilibrium domain width on these parameters leads to an exponential evolution of the domain width as shown in Fig. 1 (d) of the main manuscript and in Fig. 1 for a different Fe-wedge with 2.1 $\mathrm{ML\,mm^{-1}}$). Note that the orientation of the stripe domains is mainly dictated by the four-fold magnetocrystalline in-plane anisotropy due to the single crystallinity of the films. The effect of an in-plane anisotropy (by introducing an additional term to the domain wall energy per area) can be treated in analogy to the higher order exchange terms introduced in [1] leading to orientational anisotropy.

Supplementary Note 2
Elastic Energy of Stripe Domains

From elastic theory the corresponding energy density for meandering domain walls in a system featuring a perpendicular stripe phase is given by

$$\varepsilon_\mathrm{el} = \frac{K}{2}\left[\partial_x u + \frac{1}{2}(\partial_y u)^2\right]^2 + \frac{\mu}{2}(\partial_y^2 u)^2 + \frac{\nu}{2}(\partial_y u)^2\,, \quad (2)$$

where the function $u(x,y)$ represents the local deviations from an ideal stripe pattern, as shown in Fig. 2 (a). Without limiting generality, consider the domain walls to be parallel

to the y axis. The first term is the *compression* energy density, due to a compressive deformation $\partial_x u + \frac{1}{2}(\partial_y u)^2$ [1], either by local deviations of a single domain wall or by a variation of the domain width concerning two neighboring domain walls. The second term of Eq. (2) describes bending $((\partial_y^2 u)^2)$ of the domain walls, and is therefore called *bending* energy density. The last energy term in Eq. (2) takes into account anisotropies regarding the domain wall orientation, and is based on symmetry breaking magnetocrystalline or -elastic terms or higher order exchange terms. Hence, it is referred to as the *orientation* energy density.

The individual constants within this elastic energy model can be connected to the coupling constants Ω (dipole), Γ (exchange) and λ (effective anisotropy) in the mean-field Hamiltonian as given in Eq. (1) by [1, 3]

$$K \propto \frac{\Omega}{w_{\mathrm{D}}} \qquad \text{compression constant or rigidity} \tag{3}$$

$$\mu \propto \Omega w_{\mathrm{D}} \qquad \text{bending constant} \tag{4}$$

$$\nu \propto \frac{\Gamma}{w_{\mathrm{D}} t_{\mathrm{DW}}^3} \qquad \text{orientation constant} \tag{5}$$

where t_{DW} is the domain wall width. Note that the experimental determination of the coupling constants is extremely difficult in this kind of heterogeneous layered system (Fe/Ni/Cu(001)). Since the Fe/Ni layer grows in the fct-phase, the use of bulk exchange coupling parameters as well as the effective magnetization values of the bulk is highly inaccurate, which makes the determination of exact values of the elastic energy constants meaningless while qualitatively the theory describes the experimental observation well [6, 7].

A stripe domain state with a large domain width is more likely to exhibit corrugations due to a small compression constant K, as shown in Fig. 2 (d). In contrast, a small domain width may exhibit more undulating excitations, Fig. 2 (c), rather than local changes of the domain width, i.e. corrugations, due to a large K. This is also supported by a smaller bending constant μ for a small domain width w_{D}. Hence, in the vicinity of the SRT a more wavy domain pattern may occur with a narrower but more rigid domain width, which is known as *transverse instability* [2]. Eq. (5), however, implies that for a narrow domain configuration near the SRT the tendency towards orientation is enhanced. This is the reason why there are two scenarios for the domain pattern evolution beginning from a stripe domain phase through various other domain patterns until the magnetization direction eventually rotates into the plane as shown by Portmann et al. [6].

Supplementary Note 3
Method to determine the motion of domain walls

In order to extract fluctuations of individual domains, in the sense of tracing the motion of domain walls, the following procedure is used. At first a sequence of images is recorded. Due to vibrations in the experimental setup, the individual images of the recorded movie have to be realigned. Then we identify the domain walls by an edge detection algorithm. An example image of the result of this edge detection algorithm is shown in Fig. 3 (b), which displays solely the domain walls extracted from Fig. 3 (a). The summation of all these domain wall images, as shown in Fig. 3 (c), reveals domain regions exhibiting fluctuations and regions of domain walls which are constant in time (as far as the resolution and measurement duration are concerned). The temporal evolution of an individual domain wall can then be extracted by linescans across the domain wall of interest performed for all images in chronological order, as shown in Fig. 3 (d). As can be seen in the upper plot of Fig. 3 (d) which shows the temporal evolution of the domain wall depicted in the inset of Fig. 3 (i), the domain wall is trapped in only four positions. This means, that the local energy landscape comprises four minima. In this measurement the pixel separation of the camera corresponds to a distance of $\sim 48\,\mathrm{nm}$ on the sample. The resolution in these images was about 70-100 nm measured at different domain walls. Note, that in the lower panel of Fig. 3 (d) the fluctuations of the domain wall are mainly between two states, very close in distance.

Supplementary Note 4
Determination of orientational order

A quantification of the symmetry of the domain pattern is given by the *orientational order parameter*

$$g_n = \left| \frac{1}{N} \sum_{\mathbf{r}\in\mathrm{DW}} e^{i\,n\,\Theta_y(\mathbf{r})} \right| = \left| \langle e^{i\,n\,\Theta_y(\mathbf{r})} \rangle_{\mathbf{r}\in\mathrm{DW}} \right| \ , \tag{6}$$

where $\Theta_y(\mathbf{r})$ is the director field of the domain configuration, which refers to the angle of the domain wall (DW) at site $\mathbf{r} \in DW$ with respect to the y-axis, see Fig. 2 (a). Note, that g_n does not depend on the reference axis and is only a measure of the average deviation from a predominant orientation of the domain walls with respect to a n-fold symmetry. For instance, in the case of an ideal stripe pattern oriented along an arbitrary direction, one has

$g_n = 1$ for all n. Fig. 4 shows the evolution of g_2 for the domain patterns shown in Fig. 3 (a) of the main manuscript. For the larger domain widths the orientational order parameter is approximately equal while it decreases significantly for the pattern with 400 nm wide domains.

Supplementary Note 5
Fluctuations of a topological C-type defect

A C-type defect, which can also be considered as being composed of two strongly bound A-type defects, has two main possibilities for fluctuations. The first one is a synchronous translation of the bridge, which means that the two domain endings move simultaneously in the same direction. The other possibility is the opening of the gap between the endings, i.e. one or both endings move away from each other. However, the latter requires that the surrounding domains take part in this pattern transformation process, since otherwise the domain width of the domain between the two endings increases in size. Hence, the propensity of the system to keep the domain width as constant as possible is thereby satisfied. This is also the reason for the synchronous movement of the two endings in the former case. Both types of fluctuation of C-type defects have been observed. The synchronous, translational fluctuation of the bridge is shown in Fig. 5. The sample is the same as in the main article (Fig. 1) and the investigated region is close to the SRT, which leads to a domain width of $w_D \approx 400$ nm. Tracing the domain walls, as plotted in Fig. 5 (a), shows that the domain width (in this case of the black domain) remains almost constant. Beyond ~ 100 s the fluctuations are too fast to be resolved with the frame rate used in this experiment. The other possibility for fluctuations of a C-type defect is presented in Fig. 6. In this case the motion of the two domain endings of the C-type defect occurs in opposite directions. Hence, the black domain between these two endings becomes larger. As a result the surrounding – initially straight – domains have to bend. As can be seen from Fig. 6 (b), all four domain walls move (jump). The red dotted lines mark the two jumps of the left domain ending, which seems to be the trigger event for the domain transformation. Due to the rather high frame rate of 200 fps, a delayed response of the individual participants is revealed as shown in Fig. 6 (c). This magnified view shows the region around the second jump of the left domain ending. The rather large delay times may be caused by the unfavorable 'final' domain state of the stripe domains, since the undulated state is energetically not favorable.

Supplementary References

[1] A.B. Kashuba and V.L. Pokrovsky. Stripe domain structures in a thin ferromagnetic film. *Phys. Rev. B*, **48**, 10335-10344 (1993)

[2] M. Seul and R. Wolfe. Evolution of disorder in two-dimensional stripe patterns: "Smectic" instabilities and disclination unbinding. *Phys. Rev. Lett.*, **68**, 2460-2463 (1992)

[3] Ar. Abanov, V. Kalatsky, V. L. Pokrovsky and W. M. Saslow. Phase diagram of ultrathin ferromagnetic films with perpendicular anisotropy. *Phys. Rev. B* **51**, 1023-1038 (1995).

[4] A.B. Kashuba and V.L. Pokrovsky. Stripe domain structures in a thin ferromagnetic film. *Phys. Rev. B* **48**, 10335-10344 (1993)

[5] J. Choi, J. Wu, C. Won, Y. Z. Wu, A. Scholl, A. Doran, T. Owens and Z. Q. Qiu. Magnetic Bubble Domain Phase at the Spin Reorientation Transition of Ultrathin Fe/Ni/Cu(001) Film. *Phys. Rev. Lett.*, **98**, 207205 (2007)

[6] O. Portmann, A. Vaterlaus and D. Pescia. An inverse transition of magnetic domain patterns in ultrathin films. *Nature* **422**, 701-704 (2003)

[7] C. Won, Y. Z. Wu, J. Choi, W. Kim, A. Scholl, A. Doran, *et al.* Magnetic stripe melting at the spin reorientation transition in Fe/Ni/Cu(001). *Phys. Rev. B* **71**, 224429 (2005).
